# Indole Treatment Alleviates Intestinal Tissue Damage Induced by Chicken Coccidiosis Through Activation of the Aryl Hydrocarbon Receptor

**DOI:** 10.3389/fimmu.2019.00560

**Published:** 2019-03-26

**Authors:** Woo H. Kim, Hyun S. Lillehoj, Wongi Min

**Affiliations:** ^1^Animal Biosciences and Biotechnology Laboratory, U. S. Department of Agriculture, Beltsville Agricultural Research Center, ARS, Beltsville, MD, United States; ^2^College of Veterinary Medicine and Institute of Animal Medicine, Gyeongsang National University, Jinju, South Korea

**Keywords:** indole, CD4^+^ T cells, Treg cells, Th17 cells, chicken, coccidiosis

## Abstract

Indoles, as the ligands of aryl hydrocarbon receptor (AhR), have been shown to possess immune-modulating property in terms of the balancing between regulatory T cells (Treg) and T helper 17 cells (Th17) activities. In the present study, we examined the effects of dietary indoles, 3,3′-diindolylmethane (DIM) and indole-3-carbinol (I3C), on CD4^+^T cell population and functions in chickens. Furthermore, the effects of dietary DIM treatment on chicken coccidiosis caused by an apicomplexan parasite were investigated. Dietary treatment of healthy chickens with DIM and I3C induced increased CD4^+^CD25^+^ (Treg) cells and the mRNA expression of IL-10, while decreasing number of CD4^+^IL-17A^+^ (Th17) cells and Th17-related cytokines transcripts expression in the intestine. In addition, we explored the role of AhR in indole-treated splenic lymphocytes by using AhR antagonist and our results suggested that DIM is a ligand for chicken AhR. In chicken coccidiosis, treatment of DIM increased the ratio of Treg/Th17 cells and significantly reduced intestinal lesion although no significant changes in body weight and fecal oocyst production were noted compared to non-treated control group. These results indicate that DIM is likely to affect the ratios of Treg/Th17 reducing the level of local inflammatory response induced by *Eimeria* or facilitate repairing process of inflamed gut following *Eimeria* infection. The results described herein are thus consistent with the concept that AhR ligand modulates the T cell immunity through the alteration of Treg/Th17 cells with Treg dominance. To our knowledge, present study is the first scientific report showing the effects of dietary indole on T cell immunity in poultry species.

## Introduction

Coccidiosis which is caused by apicomplexan protozoan parasites of *Eimeria* spp. is one of the most economically important diseases affecting poultry production (1). After chickens ingest sporulated oocysts, sporozoites are released in the intestinal tract, invading intestinal epithelial cells for intracellular development. Invasion and egress of sporozoites and merozoites, which are, two major invasive form of *Eimeria* lead to the destruction of the intestinal mucosa, thus resulting in local inflammation in the intestine (2). In *E. tenella*-infected chickens, the number of CD4^+^ lymphocytes in the intestine significantly increases (3). Early studies have shown that T lymphocytes and their cytokines are essential for immunity against *Eimeria* infection in chickens (4, 5). *Eimeria* infection elicits strong IFN-γ-driven immune responses by T cells, and it plays a crucial role in control of coccidiosis (6). However, a growing body of literature implicates Th17- and Treg-related cytokines in host defense by the intestinal lymphocytes during *Eimeria* infection in chickens (7–10).

Indoles are phytochemicals that are very common in the body and diet and are abundant in Brassica (cruciferous) vegetables, including broccoli, Brussels sprouts, cabbage, and cauliflower (11). After ingestion, indole compounds such as 3,3′-diindolymethane (DIM) and indole-3-carbinol (I3C) are converted from glucosinolates, which are abundant in cruciferous vegetables (11). Both DIM and I3C are ligands for the aryl hydrocarbon receptor (AhR) and have been found to exhibit anti-inflammatory and anticancer properties through AhR activation (12, 13). AhR is a ligand-activated transcription factor recognizing a consensus xenobiotic responsive element binding site located in the upstream regulatory regions of target genes including cytochrome P450 family 1 members such as CYP1A1 and CYP1A2 (14–16). Recently, several studies have focused on activation of AhR by indoles in CD4^+^ T cell immunity; interestingly, the findings have indicated different effects on the differentiation of T cell subsets, particularly regulatory T (Treg) and T helper 17 (Th17) cells, depending on the type of indole, although the underlying mechanism is not fully established (17–21). For example, 6-formylindolo[3,2-b]carbazole (FICZ), the tryptophan photoproduct containing two indole rings, specifically induces the differentiation of Th17 cells (21–23), whereas DIM and I3C promote the generation of Treg cells and the suppression of Th17 cells (17, 18). Treg and Th17 cells are relatively newly described lineages of CD4^+^ T helper cells. Although Treg and Th17 cells share a common precursor cell (the naïve CD4 T cell) and require a common tumor growth factor (TGF)-β signal for initial differentiation, Treg cells play a role in the maintenance of T cell homeostasis and regulation of self-tolerance, whereas Th17 cells are involved in the inflammatory response by producing proinflammatory cytokines such as interleukin (IL)-17. The interplay or balance between Treg and Th17 cells is a major factor in inflammation (24).

The effects of indole compounds in chickens and the roles of Treg and Th17 cells in chicken coccidiosis have not been extensively studied. Given the ability of indoles to regulate the T cell immune response and the importance of T cell immunity in coccidiosis, in the present study, we investigated whether dietary indoles might regulate CD4^+^ T cell immunity in chicken coccidiosis. We hypothesized that DIM and I3C administered orally would activate AhR in chicken and lead to Treg-dominance, thereby decreasing the intestinal inflammatory response and preventing tissue damage.

## Materials and Methods

### Reagents and Antibodies

DIM (D9568, CAS no. 1968-05-04) and I3C (I7256, CAS no. 700-06-1) were purchased from Sigma (St. Louis, MO). Both DIM and I3C were suspended in DMSO (D2650, Sigma) for *in vitro* studies and diluted with corn oil purchased from a local market for *in vivo* studies. Concanavalin A (Con A, C5275), phorbol-12-myristate-13-acetate (PMA, P8139) ionomycin (I9657), and CH223191 (C8124) were purchased from Sigma. Antibodies (Abs) with the following specificities were used for flow cytometry: CD4-PE (CT-4), CD8-Alexa Fluor 700 (CT-8), CD3-Pacific blue (CT-3), and CD45-APC (LT40) (Southern Biotech, Birmingham, AL). The following antibodies were purified and conjugated in-house: CD25-FITC (#32) and IL-17A-FITC (1G8) (25, 26).

### Chickens

Newly hatched broiler chickens (Ross/Ross) were purchased from Longnecker's Hatchery (Elizabethtown, PA) and housed in electrically heated battery starter cages (Petersime, Gettysburg, OH). All chickens were raised in starter cages until 14 days of age and transferred to finisher cages, where they were kept until they are sacrificed. Feed and water were provided *ad libitum* under coccidian-free conditions. We used 150 birds for *in vivo E. tenella* infection study and another 12 healthy birds were used for preparation of lymphocytes from spleen and cecal tonsil. Animal husbandry followed the guidelines for the care and use of animals in agricultural research. All experiments were approved and followed by the United States Department of Agriculture (USDA)-Agricultural Research Service Beltsville Institutional Animal Care and Use Committee (protocol number: 18-019).

### Cell Culture

Chicken primary lymphocytes from cecal tonsils or spleen were isolated as previously described with modifications (8). Briefly, spleen and cecal tonsils were collected aseptically from healthy chicken and homogenized using gentleMACS Dissociator (Miltenyi Biotec, Gaithersburg, USA). The lymphocytes were purified by a Histopaque-1077(Sigma) density gradient method. Freshly purified primary lymphocytes from cecal tonsils or spleen were cultured in complete RPMI-1640 (GE Healthcare, Pittsburgh, PA) supplemented with 10% FBS (GE Healthcare), penicillin/streptomycin (10,000 unit/ml, Invitrogen, Carlsbad, CA), 50 μg/ml gentamycin (Sigma), 25 mM HEPES (Gibco, Gaithersburg, MD), and 55 μM 2-Mercaptoethanol (Gibco). For sporozoite viability test, chicken epithelial cell line (MM-CHiC clone, 8E11) was purchased and cultured in DMEM/F-12 (1:1, Sigma) supplemented with 2 mM L-glutamine (Sigma), 10 % FBS, and 10,000 unit/ml penicillin/streptomycin.

### Parasite Propagation and Preparation of Sporozoite Antigen

To obtain sporulated *E. tenella* oocysts (ARS strain) for *in vivo* study, unsporulated were purified from the feces of infected chickens, and sporulation was conducted with incubation in 2.5% potassium dichromate solution for 48 h. Sporozoites of *E. tenella* were obtained by excystation of sporulated oocysts (27). Briefly, freshly sporulated oocysts were disrupted with 0.5-mm glass beads for 5–7 s by using a Mini-beadbeater (BioSpec Products, Bartlesville, OK). The released sporocysts were purified by isopycnic centrifugation in a Percoll gradient and washed in ice-cold Hank's balanced salt solution (HBSS, Sigma), and the excystation of sporozoites was induced by treatment with 0.25% trypsin and 0.014 M taurocholic acid (Sigma) at 41°C for 90 min. The excysted sporozoites were collected, washed three times with HBSS at 3,000 × g for 10 min at 4°C and resuspended to 1.0 × 10^7^/ml in HBSS. *E. tenella* sporozoite antigen (EtSzAg) was obtained through a series of sonication and freeze and thaw cycles followed by filtration with a 0.22 μm filter. The concentration was measured with a Pierce BCA Protein Assay kit (Thermo Fisher Scientific, Frederick, MD), and samples were stored at −80°C until use.

### Intracellular Staining and Flow Cytometry

For the intracellular staining of IL-17A, lymphocytes were stimulated with PMA (10 ng/ml) and ionomycin (500 μg/ml) in complete RPMI-1640 for 4 h in the presence of golgiplug (1 μl/1 × 10^6^ cells, BD, Franklin Lakes, NJ). Cells were analyzed with a Cytoflex flow cytometer (Beckman Coulter, Brea, CA). Lymphocytes from single-cell suspensions were identified according to their light scattering properties and the CD45^+^ population. Potential doublet cells were discriminated by FSC-H/FSC-W, and dead cells were excluded by using Fixable Viability Stain 780 (BD). Treg cells and Th17 cells were designated as CD45^+^CD3^+^CD4^+^CD25^+^ and CD45^+^CD3^+^CD4^+^IL-17A^+^ cells, respectively. Unfortunately, foxp3, a signature transcription factor for Treg, has not been cloned in chickens; thus, we had to consider the CD4^+^CD25^+^ phenotype as being indicative of Treg cells (28, 29).

### Quantitative Real-Time PCR

RNA was isolated from primary lymphocytes from the cecal tonsils or spleen by using an RNeasy Isolation Kit (Qiagen, Germantown, MD), per the manufacturer's instructions, then treated with RNase-free DNase (Qiagen) and eluted in RNase-free water (Qiagen). The concentration and purity of the RNA were measured using a NanoDrop spectrophotometer (Thermo Fisher Scientific). cDNA was synthesized using random hexamer primers and a QuantiTect Reverse Transcription Kit (Qiagen). Real-time RT-PCR was performed using a Stratagene Mx3000P thermocycler (Agilent Technologies, USA) with a QuantiTect SYBR Green PCR Kit (Qiagen) and the various chicken chemokine and cytokine primers listed in Table 1. A melting curve was obtained at the end of each run to verify the presence of a single amplification product without primer dimers. Standard curves were generated using serial five-fold dilutions of cDNA to validate the amplification efficiency. The fold changes in each transcript were normalized to β-actin and are reported relative to the transcript expression in the vehicle control group or non-infected group (normalized to 1), on the basis of the comparative ΔΔCt method, as previously described (27).

**Table 1:** List of quantitative real-time RT-PCR primers used in this study.

**Target**	**Primer and sequence**	**References**
IL-10	(For) 5′-ACATCCAACTGCTCAGCTCT-3′	(30)
	(Rev) 5′-ATGCTCTGCTGATGACTGGT-3′	
IL-17A	(For) 5′-GAGAAGAGTGGTGGGAAAG-3′	(31)
	(Rev) 5′-TCTACAAACTTGTTTATCAGCAT-3′	
IL-17F	(For) 5′-TGAAGACTGCCTGAACCA-3-3′	(31)
	(Rev) 5′-AGAGACCGATTCCTGATGT-3′	
IL-21	(For) 5′-CAACTTCACCAAAAGCAATGAAAT-3′	(32)
	(Rev) 5′-ATCCATCCCCAGGGTTTTCT-3′	
IL-22	(For) 5′-TGTTGTTGCTGTTTCCCTCTTC-3′	(33)
	(Rev) 5′-CACCCCTGTCCCTTTTGGA-3′	
CYP1A4	(For) 5′-CCGTGACAACCGCCCTGTCC-3′	(34)
	(Rev) 5′-GAGTTCGGTGCCGGCTGCAT-3′	
CYP1A5	(For) 5′-GGACCGTTGCGTGTTTAT-3′	(35)
	(Rev) 5′-CTCCCACTTGCCTATGTTTT-3′	
IL-1β	(For) 5′-TGGGCATCAAGGGCTACA-3′	(31)
	(Rev) 5′-CGGCCCACGTAGTAAATGAT-3′	
IL-6	(For) 5′-CAAGGTGACGGAGGAGGAC-3′	(31)
	(Rev) 5′-TGGCGAGGAGGGATTTCT-3′	
CXCLi2	(For) 5′-GGCTTGCTAGGGGAAATGA-3′	(31)
	(Rev) 5′-AGCTGACTCTGACTAGGAAACTGT-3′	
TL1A	(For) 5′-CCTGAGTTATTCCAGCAACGCA-3′	(36)
	(Rev) 5′-ATCCACCAGCTTGATGTCACTAAC-3′	
JAM2	(For) 5′-AGCCTCAAATGGGATTGGATT-3′	(37)
	(Rev) 5′-CATCAACTTGCATTCGCTTCA-3′	
ZO1	(For) 5′-CCGCAGTCGTTCACGATCT-3′	(37)
	(Rev) 5′-GGAGAATGTCTGGAATGGTCTGA-3′	
β-actin	(For) 5′-CACAGATCATGTTTGAGACCTT-3′	(38)
	(Rev) 5′-CATCACAATACCAGTGGTACG-3′	

### *Eimeria tenella* Infection Model

For *in vivo* study, One-hundred-fifty 1-day-old birds were randomly distributed into five groups (*n* = 30): non-infected control (NI), non-infected, DIM treated (NIDIM), *E. tenella*-infected (ET), *E. tenella*-infected, vehicle treated (ETVH), and *E. tenella*-infected, DIM treated (ETDIM). The schematic outline of *in vivo* study is shown in Figure 4A. The chickens in ET, ETVH and ETDIM groups were orally infected with *E. tenella* sporulated oocysts (1 × 10^4^/bird) at 7 days old and the other groups were given HBSS as a control. The chickens in the NIDIM and ETDIM groups were treated every other day (starting at 5 days old) with DIM (200 mg/kg) and the other groups were given corn oil as a vehicle control by oral gavage until the end of experimental period (20 days old). Body weight gain (BWG) was measured at 0 and 13 days post infection (DPI) (*n* = 15). Cecal tissues were collected from four chickens of each group at 1, 4, 7, 10, and 13 days post infection (DPI) to extract RNA, and the expression of Treg- and Th17-related mRNAs was analyzed (*n* = 4). Five chickens from each group were randomly selected for gut lesion scoring in the cecum at 7 DPI (*n* = 5). Lesion scores were evaluated by three independent observers based on scoring techniques previously described (39). Each chicken received a numerical value from 0 to 4. Same cecal samples were used for histological examination (*n* = 5). Briefly, the tissues were fixed in 4% paraformaldehyde (Sigma), and paraffin blocks were prepared, microtome sections were made, and sections were stained using hematoxylin and eosin. The sections were examined for intestinal structure, parasites and infiltration of inflammatory cells using an Eclipse 80i microscope (Nikon, Japan). To count fecal *Eimeria* oocyst shedding, collection of feces from three cages of each group was started at 5 DPI until 9 DPI (*n* = 3), and the number of *E. tenella* oocyst in the feces was calculated, as previously described, by using a McMaster counting chamber (Marienfeld-Superior, Germany) (40). The total number of oocysts was calculated according to the following formula: total oocysts = oocysts counted × dilution factor × fecal sample volume/counting chamber volume. The values were converted as oocyst per gram of feces.

### Sporozoite Viability Test

To assess the viability of sporozoites, purified sporozoites were incubated with DIM (0–500 μM) for 24 h, the viability was measured using CyQuant direct cell proliferation assay (41). To infect sporozoites into chicken epithelial cell line (8E11), purified sporozoites were stained with carboxyfluorescein succinimidyl ester (CFSE, Thermo Fisher Scientific) according to manufacturer's instructions. 8E11 was cultured in DMEM/F12 supplemented with 10% FBS, penicillin/streptomycin, and 25 mM HEPES and the sporozoites was infected at a multiplicity of infection of 1.0 (sporozoite/cell ratio of 1:1). Free sporozoites were washed after 3 h incubation and new media was replaced. After further incubation for 21 h, the number of sporozoites was measured at 485/528 nm.

### Statistical Analysis

The data were analyzed using Prism Version 5.01 (GraphPad Software, La Jolla, CA). The normality of each data was tested by Kolmogorov–Smirnov test. Parametric tests were used to compare between groups with one-way ANOVA and Dunnett's multiple comparison test and non-parametric tests were conducted with Kruskal–Wallis test and Dunn's multiple comparison test. The data are expressed as the mean ± standard error for parametric analysis and median with interquartile range for non-parametric analysis and the differences were considered significant at *p* < 0.05 or *p* < 0.01.

## Results

### Increased Treg Cells and Th17/Treg Ratio in Indole-Treated Chickens

**Figure 1:**
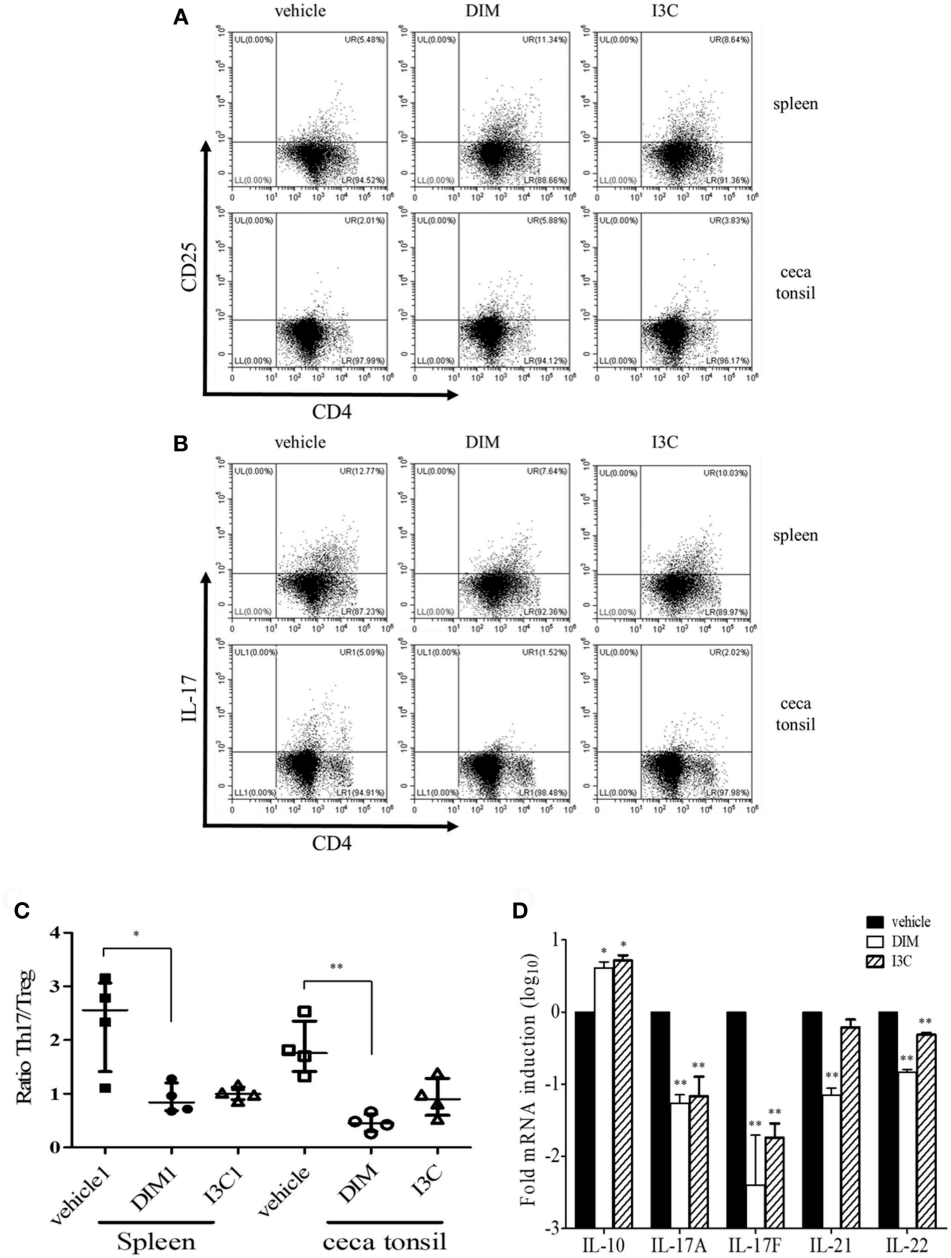
Effects of indoles to induce Treg cells in indole-treated chickens. Two-weeks-old chickens were given orally corn oil as a vehicle control, DIM (200 mg/kg), or I3C (200 mg/kg) for 14 days on a daily basis. Lymphocytes were isolated from the spleen and cecal tonsils, and stained with CD3, CD45, CD4, and CD25, or CD3, CD45, CD4, and IL-17A for analyzing Treg **(A)** and Th17 **(B)** populations, respectively, and gated CD4+ T cells. Cells were stimulated with PMA and ionomycin for the determination of Th17 cells. Data show the representative staining from two independent experiments. **(C)** The ratio of Th17/Treg cells in the spleen and cecal tonsils. The data represent median with interquartile range after Kruskal-Wallis test with Dunn's multiple comparison test. **(D)** RNA was isolated from cecal tonsils and used for real-time qPCR to measure Treg- (IL-10) and Th17-related (IL-17A, IL-17F, IL-21, and IL-22) mRNA expression. The data represent the mean ± SE from two independent experiments. ^*^p < 0.05 and ^**^p < 0.01 were considered statistically significant compared to the vehicle control in each sample or target.

To determine the dietary effects of indole treatment on CD4^+^ T cells in healthy chickens, we firstly investigated the frequencies of CD4^+^CD25^+^ (Treg) and CD4^+^IL-17A^+^ (Th17) cells from the spleen and intestine after oral treatment of either DIM or I3C. In the indole-treated groups compared with the vehicle control group, Treg cells were significantly higher, whereas the treatment with indoles induced a decrease in Th17 cells in both the spleen and cecal tonsils (Figures 1A,B). The ratio of Th17/Treg cells decreased in both indole-treated groups (Figure 1C). There was no significant difference in the frequencies of Treg or Th17 cells between the treatments with DIM and I3C. Furthermore, the real-time qPCR results showed that indoles increased the mRNA expression of the Treg-related cytokine IL-10 and decreased Th17-related cytokines such as IL-17F, IL-21, and IL-22 in cecal tonsils (Figure 1D).

### The *in vitro* Effects of Indoles on Treg Cells and Aryl Hydrocarbon Receptor

**Figure 2:**
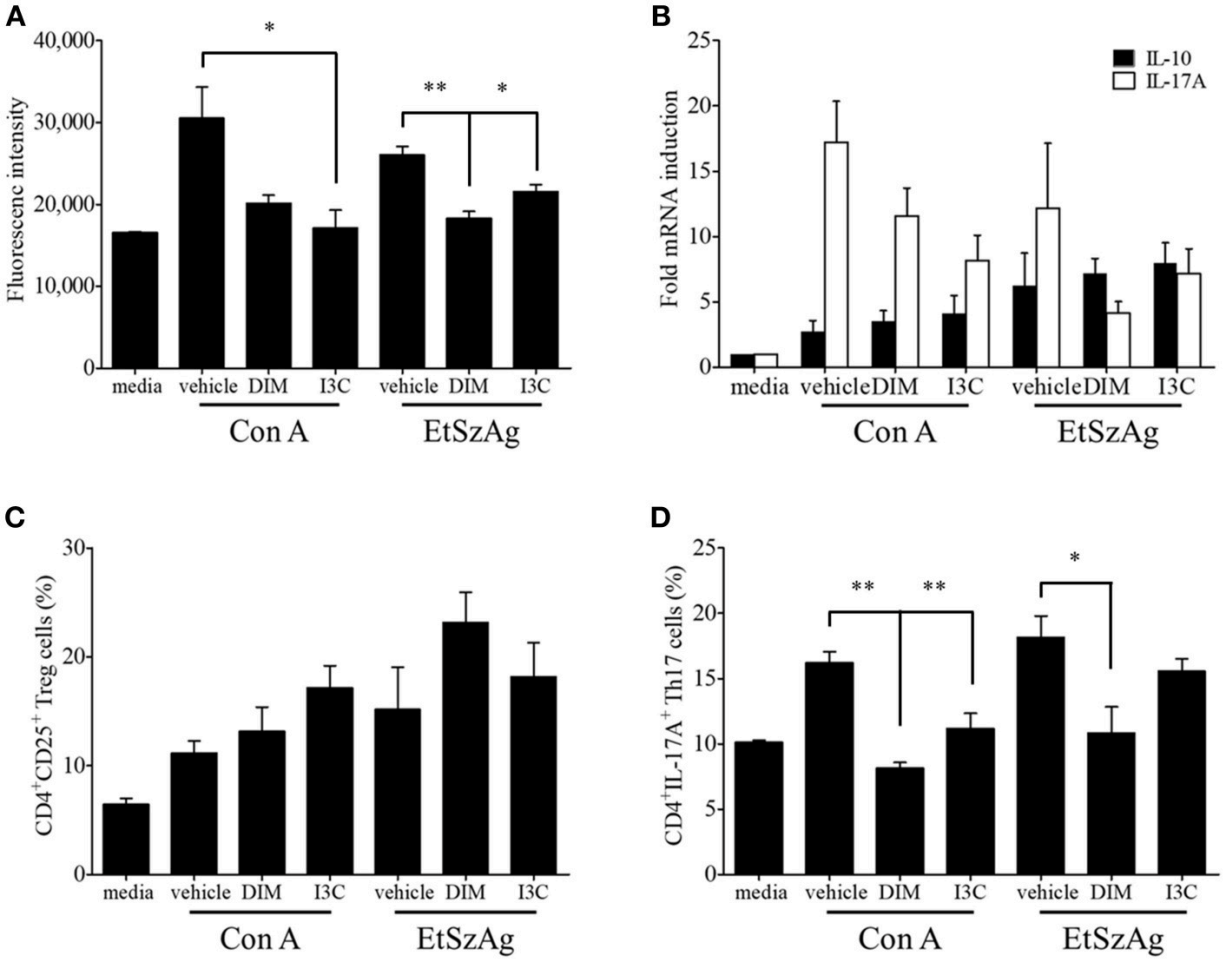
Effects of indoles to induce Treg cells *in vitro*. **(A)** Lymphocytes from spleen of healthy chickens were isolated and stimulated with DMSO as a vehicle control, DIM (100 μM), or I3C (100 μM) in the presence of Con A (10 μg/ml) or EtSzAg for 24 h and the proliferation was measured by CyQuant direct cell proliferation assay. **(B)** The mRNA expressions of Treg- and Th17-related cytokine were measured after 24 h by real-time qPCR. The frequency of Treg **(C)** and Th17 cells **(D)** were analyzed by flow cytometry. The data represent the mean ± SE from two independent experiments. ^*^p < 0.05 and ^**^p < 0.01 were considered statistically significant compared to the vehicle control of each treatment.

**Figure 3:**
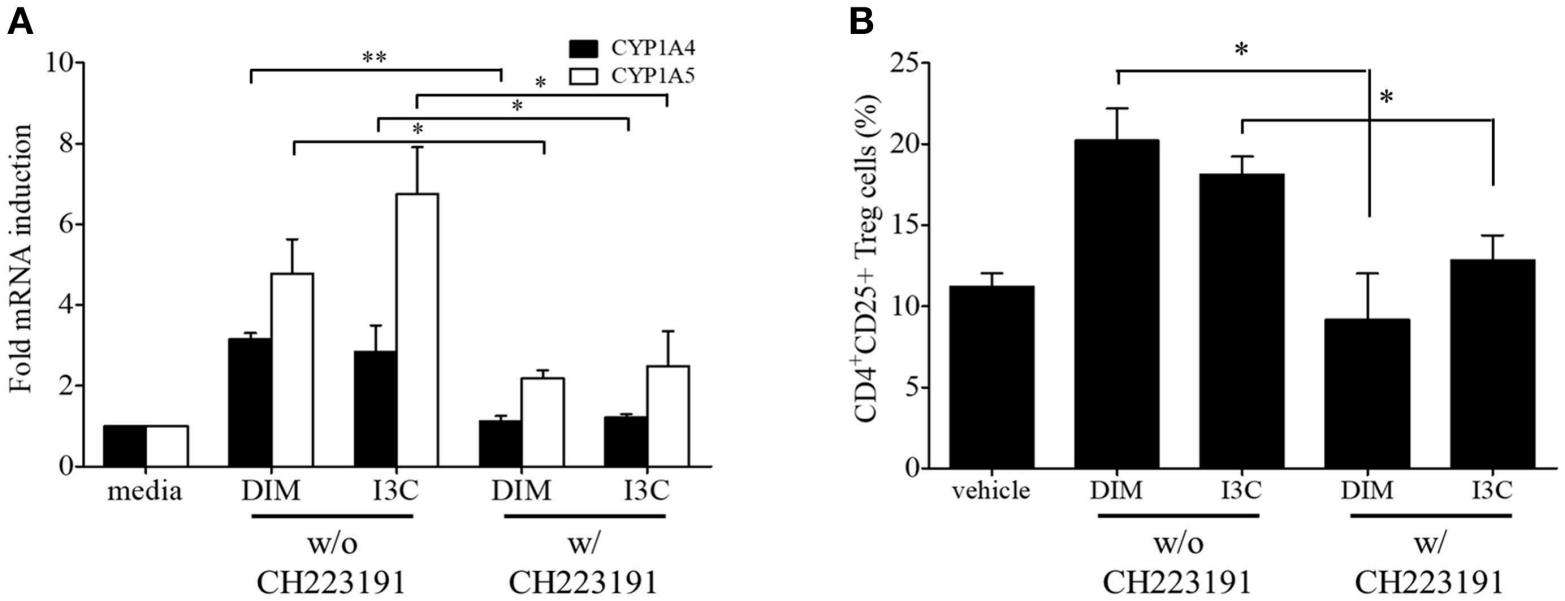
Regulation of indoles by AhR. Lymphocytes from cecal tonsils were isolated and stimulated with DMSO as a vehicle control, DIM (100 M/ml), or I3C (100 M/ml) in the absence of presence of specific AhR antagonist, CH223191 for 24 h. **(A)** The mRNA expressions of CYP1A4 and CYP1A5 were measure by real-time qPCR. **(B)** The frequency of Treg cells were analyzed by flow cytometry. The data represent the mean ± SE from two independent experiments. ^*^p < 0.05 and ^**^p < 0.01 were considered statistically significant compared to the samples.

To validate *in vivo* findings on the role of indoles on CD4+ T cell subsets, we performed *in vitro* experiments to investigate the effects of indole treatment on Treg cells in chicken lymphocytes. Chicken splenic lymphocytes were purified and stimulated with Con A or EtSzAg in the presence or absence of both indoles for 72 h. The data indicated that cell proliferation induced by Con A was inhibited by both DIM or I3C, as compared with the results for Con A-stimulated cells. Interestingly, indole treatment also inhibited the proliferation induced by EtSzAg, thus suggesting a role in coccidiosis in chickens (Figure 2A). We further investigated the mRNA expression of Treg-related (IL-10) or Th17-related (IL-17A) cytokine mRNAs in those cells. Both indole treatments significantly up-regulated expression of IL-10 while down-regulating IL-17A (Figures 2B,C). In agreement with data from *in vivo* experiments, the proportion of Treg cells in *in vitro* assays was increased in splenic lymphocytes incubated with DIM or I3C (Figure 2D). To test the hypothesis that indoles are ligands for AhR and can cause AhR activation in chickens as in mammals (42), we determined the mRNA expression levels of the chicken cytochrome P-450 enzymes CYP1A4 and CYP1A5, which are orthologous to mammalian CYP1A1 and CYP1A2, in indole-treated cecal tonsil lymphocytes. Both are AhR-regulated genes and markers of AhR activation (43). As shown in Figure 3A, the expression of CYP1A4 and CYP1A5 increased after treatment with DIM or I3C, and normal expression was restored in the presence of the AhR-specific antagonist CH223191 (Figure 3A). Furthermore, the frequency of Treg cells was higher in DIM-treated cecal tonsil lymphocytes than in non-treated cells in the presence of CH223191 (Figure 3B).

### Effects of DIM on Treg and Th17 Cells in *E. tenella* Infection

**Figure 4:**
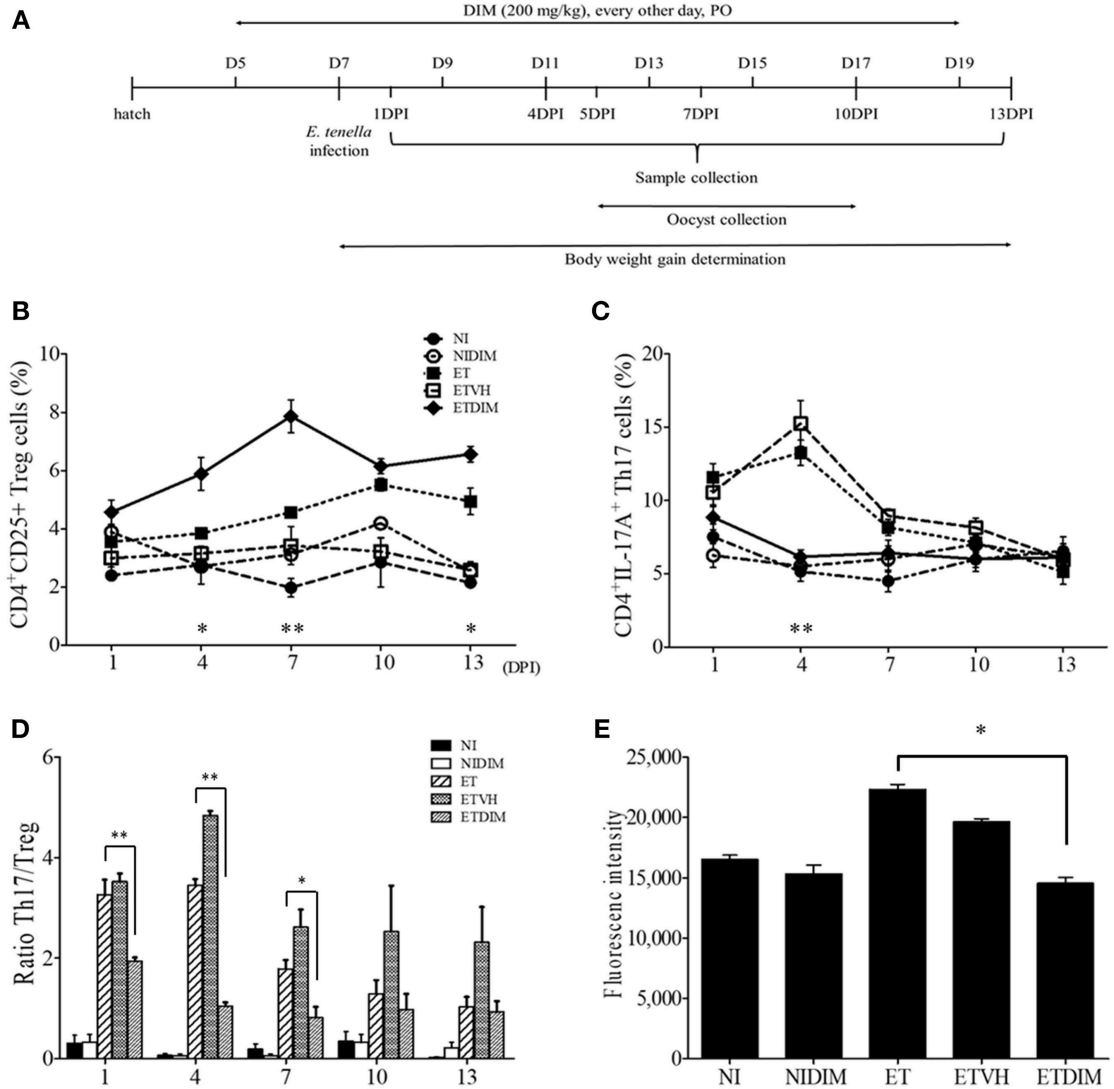
Effect of dietary DIM on intestinal T cells in *E. tenella* infection. **(A)** Schematic outline of the *in vivo* experimental design. The frequencies of Treg **(B)** and Th17 cells **(C)** of cecal tonsil lymphocytes were analyzed at indicated DPIs by flow cytometry. ^*^p < 0.05 and ^**^p < 0.01 were considered statistically significant compared between the ET and ETDIM groups at each time point. **(D)** The ratio of Th17/Treg cells in and cecal tonsils. **(E)** Lymphocytes were stimulated with Con A (10 μg/ml) and the proliferation of cecal tonsil lymphocytes was measured by CyQuant direct cell proliferation assay in EtSzAg-activated cells. ^*^p < 0.05 and ^**^p < 0.01 were considered statistically significant compared to the samples.

Because our findings indicated that indoles induced Treg cells while suppressing Th17 cells in chickens, and Th17 is known to play a pathological role in coccidiosis (7, 44), we designed the *in vivo* experiment to determine the effect of DIM on the regulation of CD4^+^ T cells in *E. tenella* infection in chickens (Figure 4A). Treatment of chickens with DIM, compared with vehicle control, induced a significant increase in intestinal Treg cells while decreasing Th17 cells. Compared with the ET group, the ETDIM group showed an increase in Treg cells at from 4 DPI, and Treg cells remained elevated until the end of experiment, whereas the decrease in Th17 cells was seen only early in infection, such as at 4 DPI (Figures 4B,C). The ratio of Th17/Treg cells exhibited significant decrease at 1, 4, and 7 DPI (Figure 4D). We also found that DIM inhibited the proliferation of lymphocytes. As shown in Figure 4E, the ETDIM group showed lower proliferation than the other groups during the re-activation of the lymphocytes with DIM *in vitro*. Notably, the NIDIM group did not show any significant inhibition of proliferation, thus suggesting that the inhibitory effect of DIM is much stronger when the lymphocytes are pre-activated with *Eimeria* antigen.

### mRNA Expression of Treg- and Th17-Related Genes in DIM-Treated Chickens

**Figure 5:**
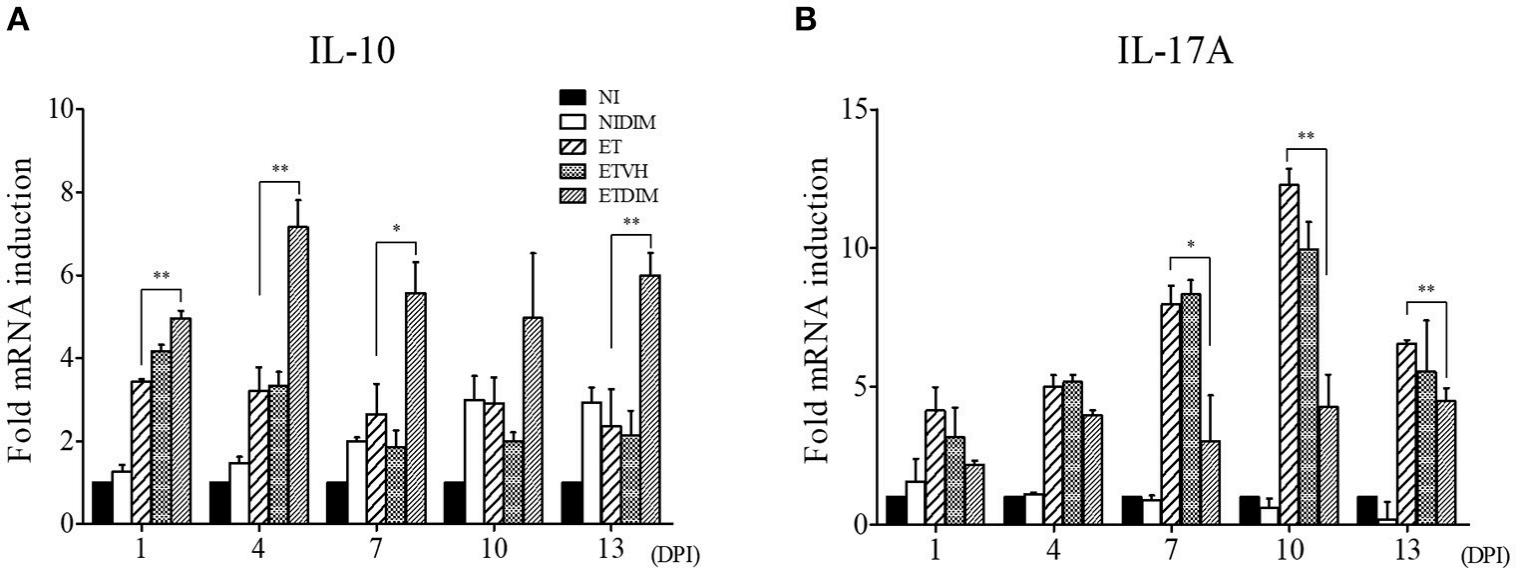
Effect of dietary DIM on mRNA expressions of Treg- and Th17-related genes in *E. tenella* infection. The mRNA expressions of IL-10 **(A)** and IL-17A **(B)** were measure in cecal tonsils by real-time qPCR. The data represent the mean ± SE from two independent experiments. ^*^p < 0.05 and ^**^p < 0.01 were considered statistically significant compared to the samples.

To determine the cytokines involved in T cell regulation by DIM, we carried out real-time qPCR to measure the mRNA expression of Treg- and Th17-related genes in T cells. In agreement with our *in vitro* findings, the expression of IL-10 increased after treatment of DIM. Following *E. tenella* infection, IL-10 expression increased in the ET group and the increased IL-10 expression in the ET groups persisted until the end of the experiment. Compared with DIM, ETDIM induced greater IL-10 expression after 1 DPI, thus suggesting that DIM induces more Treg cells in coccidiosis (Figure 5A). However, the expression of Th17-related cytokines was generally down-regulated in the DIM and ETDIM groups (Figure 5B). Interestingly, we found high expression of IL-17A in the ET group between 7 and 13 DPI, but the increases in expression of these cytokines were diminished in the ETDIM group (Figure 5B). These results suggest that *E. tenella* infection induces Th17 cells at a later stage of infection, and the suppression of Th17 cells by DIM seems to depend on the level of Th17 cytokine.

### Effects of DIM on Growth Performance, Oocyst Production, and Intestinal Lesions

**Figure 6:**
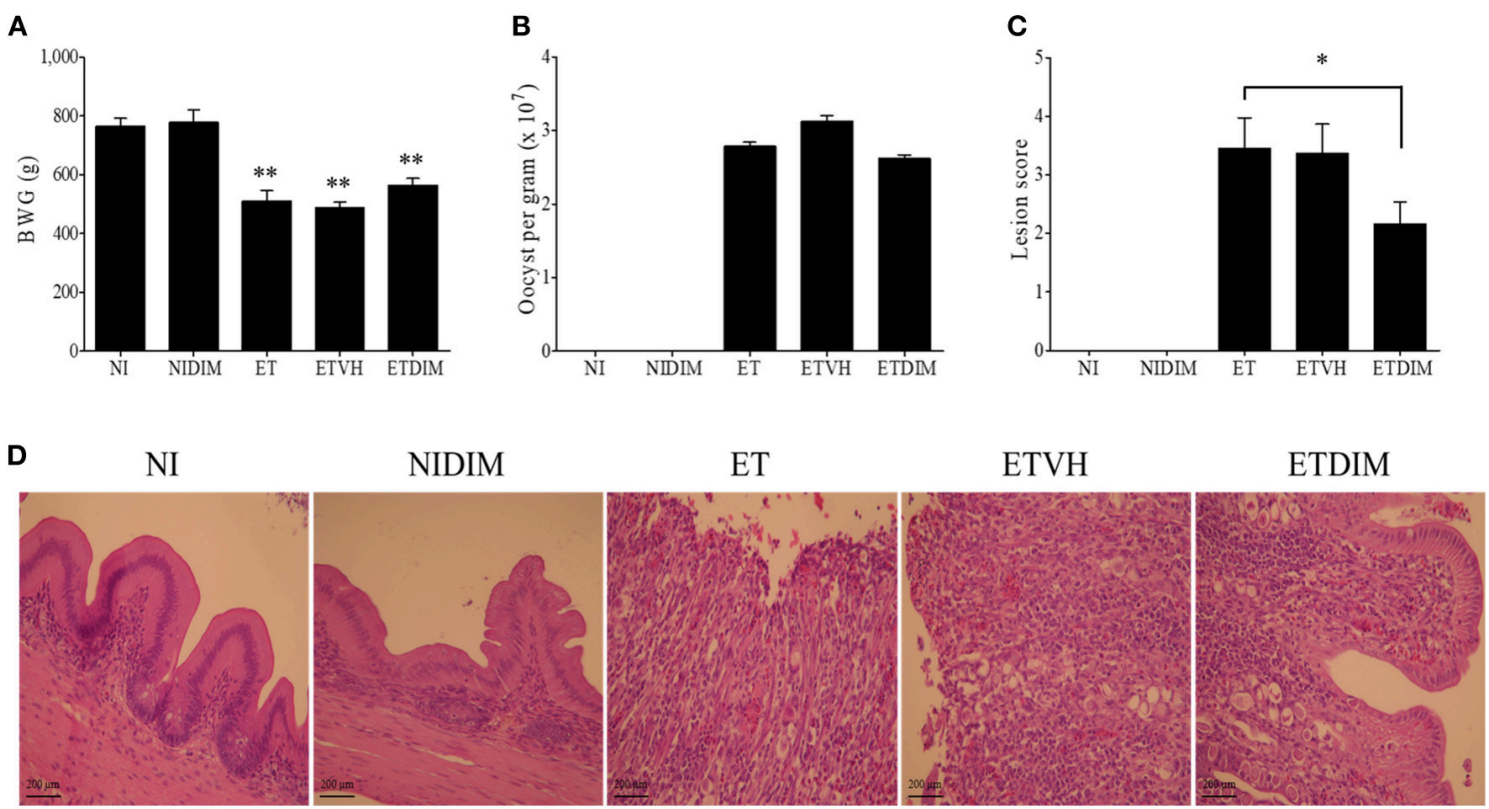
Effect of dietary DIM on growth performance, oocyst production and intestinal lesions in *E. tenella* infection**. (A)** Body weight gain was measured from 0 to 13 DPI (*n* = 15). **(B)** Fecal oocysts were collected and pooled from 5 to 9 DPI and counted using McMaster counting chamber (*n* = 15). ^*^p < 0.05 and ^**^p < 0.01 were considered statistically significant compared to the samples. **(C)** Lesion score was determined from cecum at 7 DPI (*n* = 5). **(D)** Histological sections prepared from the cecum (X400).

Next, we examined the effects of dietary DIM on BWG, oocyst production, and intestinal lesions in *E. tenella*-infected chickens. The NIDIM group showed no difference in BWG compared with the NI group, thus indicating that DIM has no effect on growth performance. Furthermore, the ETDIM and ET groups showed comparable BWG (Figure 6A). In agreement with the BWG data, the ETDIM group, compared with the ET group, did not exhibit significantly less oocyst shedding from feces (Figure 6B). Interestingly, however, the severity of intestinal lesions was significantly lower in the ETDIM group than the ET group (Figure 6C). In H&E staining, the NI and NIDIM groups displayed a normal structure and no visible changes. In all ET groups, there was structural disorder, epithelial loss, and inflammatory cell infiltration; however, the ETDIM group showed less severity in terms of abnormality of villi structure and inflammatory cell number (Figure 6D). These has been validated by our histological finding that showed the gross morphological changes in the cecum of ETDIM group showed less hemorrhage in the mucosa and less watery ingesta mixed with mucus than did the ET group (data not shown).

### mRNA Expression of Proinflammatory Genes in DIM-Treated Chickens

**Figure 7:**
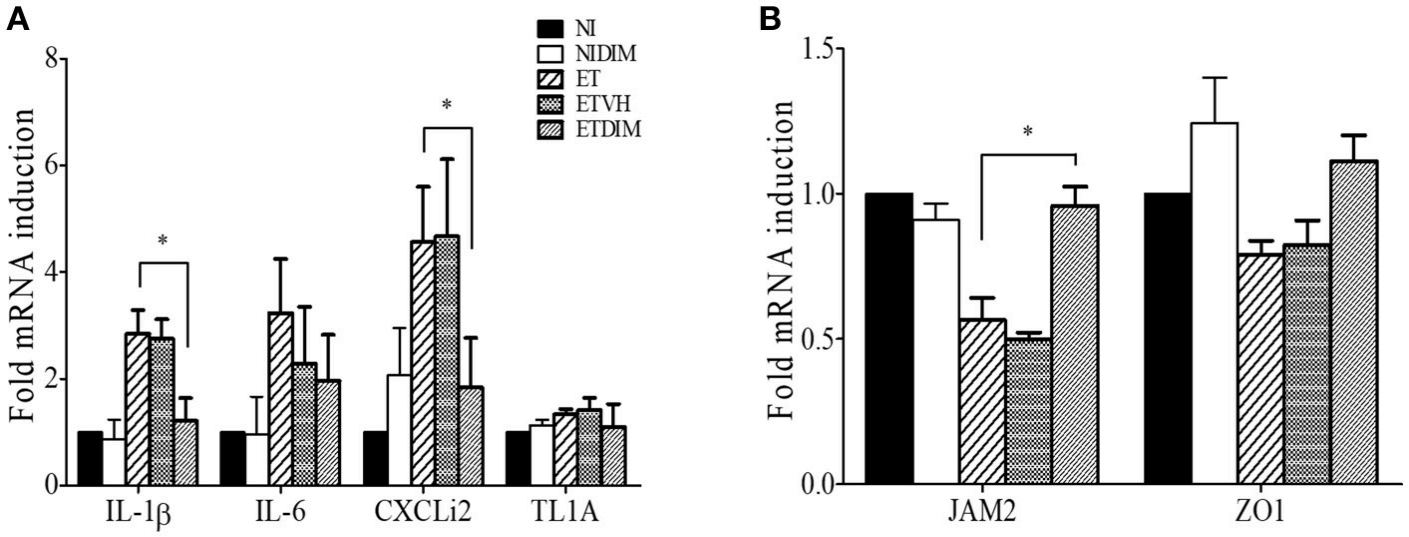
Effect of dietary DIM on mRNA expressions of proinflammatory and tight junction protein genes in *E. tenella* infection. The mRNA expressions of proinflammatory genes; IL-1β, IL-6, CXCLi2, and TL1A **(A)** and tight junction protein gene; JAM2 and ZO1 **(B)** were measure in cecum by real-time qPCR. ^*^p < 0.05 and was considered statistically significant compared to the samples.

From the findings from our *in vivo* experiments, we hypothesized that DIM treatment decreases inflammation in the intestine through the regulation of Treg and Th17 cells. To this end, we determined the mRNA expression profiles of proinflammatory genes in intestinal tissues by using real-time qPCR. As expected, *E. tenella* infection induced robust expression of proinflammatory genes such as IL-1β, IL-6, and CXCLi2 (a homolog of mammalian CXCL8) but not TNFSF15 (TL1A, a functional homolog of mammalian TNF-α) (36). Compared with the ET group, the ETDIM group showed lower expression of those genes (Figure 7A). Next, we measured the expression of tight junction proteins such as JAM2, and ZO1 to determine whether DIM treatment is involved in intestinal barrier function. Following *E. tenella* infection, mRNA expression of JAM2, and ZO1 significantly decreased, but their expression was restored to a much greater extent in the ETDIM group than the ET or ETVEH groups (Figure 7B). Together, these results suggest that treatment of DIM in coccidiosis may be beneficial to reduce intestinal inflammation and to help to restore the damage from coccidiosis.

### Direct Effect of DIM on *E. tenella*

**Figure 8:**
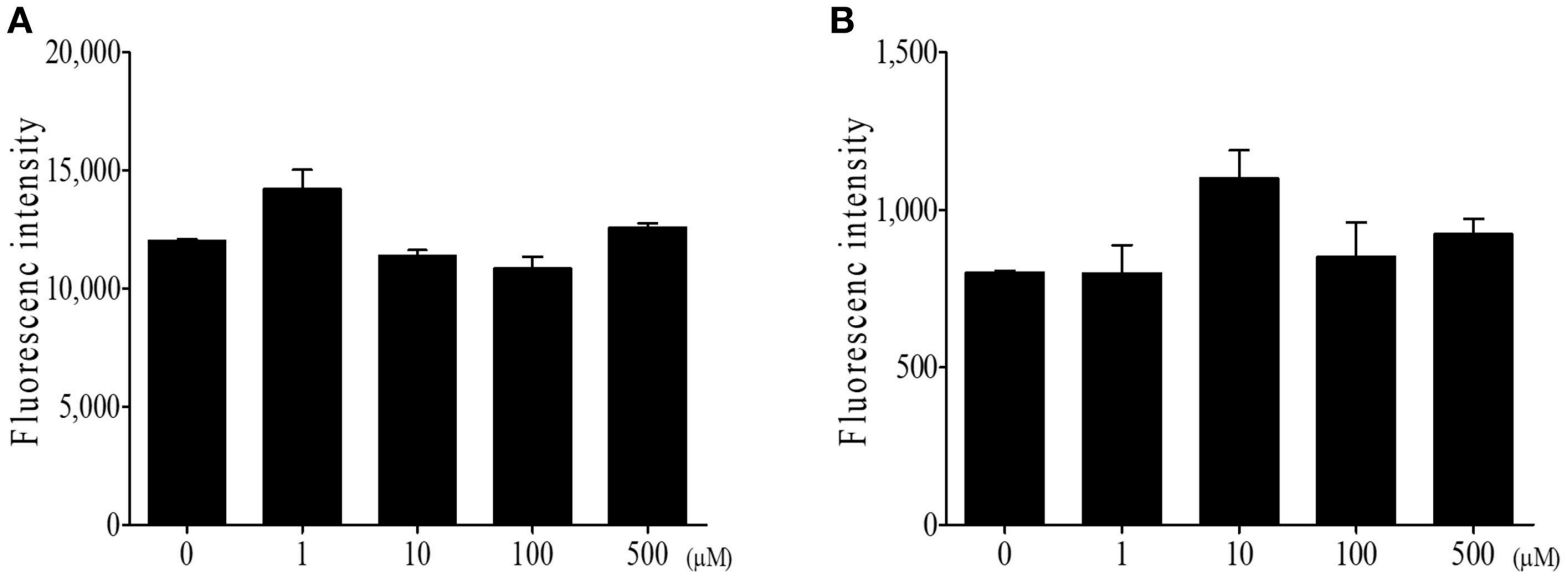
Direct effect of DIM on *E. tenella* parasite**. (A)** DIM was incubated with *E. tenella* sporozoites for 24 h and measured viability using CyQuant direct cell proliferation assay. **(B)** *E. tenella* sporozoites were stained with CFSE and infected to intestinal epithelial cells (8E11). Following infection, DIM was treated and fluorescence was measured. The data represent the mean ± SE from two independent experiments.

Finally, we determined whether DIM has any direct activity against *Eimeria* sporozoites, an invasive form of parasites. First, we incubated *E. tenella* sporozoites with various concentrations of DIM. Figure 8A shows that DIM had no effect on sporozoites viability. Second, we infected sporozoites to the chicken epithelial cell line 8E11, then treated DIM to the cells harboring sporozoites. DIM treatment did not alter the viability of sporozoites inside of cells (Figure 8B). It suggests that the beneficial effects of DIM on *E. tenella* infection are not associated with its activity against parasite itself.

## Discussion

I3C is derived from cruciferous vegetables, which contain abundant indoles. When digested, it produces several biologically active I3C oligomers such as DIM. Earlier studies of dietary indole derivatives have focused on their anti-cancer effect, given that they have been shown to reduce the risk of cancer (45–47). Indoles have been shown to induce antioxidant activity and apoptosis of cancer cells, and to regulate hormone metabolism (48–51). Recently, several studies have reported that indoles also have immunoregulatory properties, especially on T cells. For example, Singh et al. (17) have reported that dietary indoles suppress the delayed-type hypersensitivity (DTH) response through the regulation of Treg and Th17 cells in mice. Dietary supplementation of indoles also has been shown to suppress neuroinflammation by induction of reciprocal differentiation of Treg and Th17 cells in experimental autoimmune encephalomyelitis (EAE) mice (18). These abilities of indoles to modulate the immune response are related to AhR signaling (21). AhR was first discovered as a transcription factor mediating the toxicity of chemicals such as 2,3,7,8-tetrachlorodibenzo-*p*-dioxin (TCDD) (52). Recent studies have suggested that AhR activation plays diverse roles in cellular function including the regulation of the immune system (53, 54). Interestingly, there are two types of AhR found in chickens, AhR1 and AhR2, and AhR1 is the dominant form in cormorants (*Phalacrocorax carbo*) (55). In the chicken intestine, both mRNA were expressed; however further studies will be required to elucidate their role in T cell regulation (56). Moreover, there is a distinct set of cytochrome P450 family members in chickens: CYP1A4 and CYP1A5. On the basis of their amino acid sequences, they can be classified in the CYP1A family, but both are more like CYP1A1 than CYP1A2 (57). Because they are induced by AhR activation in chickens (34, 58), we identified their expression levels after DIM treatment in cecal tonsil lymphocytes. Inhibition assays using AhR-specific inhibitor confirmed that DIM and its precursor I3C induce AhR activation in chickens. Unfortunately, we did not find any evidence of an effect of chicken CYP1As on T cell regulation, thus suggesting that the mechanism of AhR activation in modulating Treg and Th17 cells may differ from the one that mediating the toxicity of environmental toxins. Quintana et al. (21) have identified an evolutionarily conserved binding site for AhR in the foxp3 gene and three non-evolutionarily conserved AhR-binding sites in the promoter regions of foxp3 genes in zebrafish, mice, and humans. Their subsequence studies proved that AhR controls foxp3 expression, which induces the generation of Treg cells. Another report has explained the possible AhR activation mechanism. Singh et al. (17) have investigated microRNA profiles in DTH mice treated with several AhR ligands and found that several microRNAs targeting foxp3 and IL-17 mRNA are important in regulating Treg and Th17 cells. The activation of AhR to regulate T cells is dependent on the type of AhR ligands. For example, TCDD induces Treg cells in EAE mice to reduce the EAE score, whereas FICZ increases Th17 cell differentiation and the severity of EAE (21, 59). DIM, an AhR ligand that we used in this study, has therapeutic effects in oxazolone-induced colitis and mBSA-induced DTH in mice through Treg cell induction and suppression of Th2 or Th17 cells (17, 60). In chickens, compared to mammals, very little is known about the effect of dietary indoles as AhR ligands on the immune response. In this study, we provide the first demonstration in chickens of how AhR ligand modulates T cells in terms of Treg and Th17 cells. As we expected, DIM and I3C increased the number of CD4^+^CD25^+^ cells in chicken lymphocytes *in vivo* as well as *in vitro*, thus suggesting that they have comparable effects to those in mammals. Regarding the Th17 cells, the data showed a decrease in CD4^+^IL-17A^+^ cells, but to a lesser extent than the increase in intestinal Treg cells. Moreover, the expression of each lineage of Th cell-related cytokines was consistent with data obtained from flow cytometry. The anti-inflammatory cytokine IL-10 showed increased expression in DIM-treated groups, whereas the expression of the IL-17A, IL-17F, IL-21, and IL-22 was downregulated in the DIM-treated groups. These data suggest that the cytokine profile for each Th lineage is likely to overlap in chickens and mammals.

We further investigated the effects of indole in regulating Treg and Th17 cells in coccidiosis. Coccidiosis caused by *Eimeria* spp. induces an inflammatory response in parasitized intestinal tissues (61). Profiling of cytokines in coccidiosis revealed that most T cell cytokines are increased along with the inflammation in the intestine (62, 63). Moreover, our previous studies have indicated that Th17-related cytokines such as IL-17A and IL-17F, and IL-17 receptor signaling are involved in inflammation induced by coccidiosis, although the predominant protective response in coccidiosis is considered to be an IFN-γ-related Th1 response (6, 8, 31, 64). We investigated the changes in Th1 cells as CD4^+^IFN-γ^+^ cells in *E. tenella* infection. Initially, we expected that DIM would also affect the Th1 response, because several studies have shown that AhR agonists can also modulate the differentiation of Th1 cells (65, 66). As shown in Figure S1, Th1 cells were highly induced at 1 and 4 DPI following *E. tenella* infection although they did not change as much as Treg and Th17 cells in the DIM-treated groups. Therefore, Th1 response may play a role during the early phase of coccidiosis by initiating local inflammatory response started by the host cell invasion of sporozoites of *Eimeria* with subsequent intracellular development in early to intermediate phase. Compared to the NI group, Treg and Th17 cells in the ET groups were induced in a later phase, thus suggesting that they might be involved in tissue recovery from the damage induced by Th17 responses or have an important function in gut homeostasis. Several studies have reported that chicken IL-17A plays a pathogenic role in *Eimeria* infection (7, 44). Likewise, in the ETDIM group, which showed a lower degree of intestinal lesions, Th17 cells were downregulated compared with their levels in the NI or DIM group, and it is likely Th17 cells are involved in pathogenicity or inhibiting recovery from inflammation. At the same time points, Treg cells showed increased populations in the ETDIM group, thus indicating that DIM increased the Treg populations and decreased the inflammation in the parasitized intestine. IL-10 has been considered to play an important role to evade host immune response in coccidiosis. One possible mechanism is that coccidial parasites have evolved to stimulate Treg cells to express IL-10 and it helps parasites to facilitate invasion and survival in chickens through the suppression of protective response mediating IFN-γ-expressing Th1 cells. Using two inbred lines of chicken differing in their resistance or susceptibility to *Eimeria* infection, it is revealed that the expression of IL-10 was the major difference between those two lines. The expression of IL-10 was highly induced in susceptible line of chickens among the genes related to different helper T cell lineages such as IFN-γ for Th1, IL-4 for Th2, and IL-10 and TGF-β for Treg cells while it is suppressed in age-matched resistant line (10). The administration of IL-10 antibody in *Eimeria*-infected chicken showed improved growth rate compared to control antibody group but it did not influence fecal oocyst production (67, 68). These results are indicating that the regulation of protective immune response to *Eimeria* spp. by Treg cells is critical and IL-10 contributes to pathogenesis in coccidiosis. In the current study, on the other aspect of Treg cells, we found another role of Treg cells that involves in anti-inflammatory response through suppress inflammatory Th17 cells. It is thought that the anti-inflammatory Treg cells could be participate in the self-limiting mechanism of *Eimeria* spp. that prevents the collateral intestinal damage caused by exaggerated inflammation. Other evidences of reduced intestinal inflammation we found in this study was the expression of proinflammatory genes and tight junction protein. Chicken IL-17A and IL-17F have known to induce proinflammatory cytokines such as IL-1β, IL-6, and CXCLi2 as in mammals (31) thus decrease of Th17 cytokines might involve the anti-inflammatory process in the intestine. As a marker of intestinal integrity, tight junction protein play an important role in the regulation of intestinal permeability by sealing the paracellular space between intestinal epithelial cells (69). The recovery of tight junction protein expressions was induced by dietary treatment of DIM and it might be associated with that mammalian IL-17A and IL-17F reported to disrupt the distribution of tight junction protein (70).

As the main source of indoles in cruciferous vegetables, Glucosinolates (GLS) have been used to measure the biologically active constituent. McNaughton and Mark reported GLS content of various cruciferous vegetables (71). It varies depending on the species (e.g., cress for the highest GLS content as 389 mg/100 g while the lowest content for Pe-tsai chinese cabbage; 20 mg/100 g) and there is large variation in the values reported for the same vegetable by different studies (71, 72). Since the US Food and Drug Administration permitted the use of claims acknowledging the relationship between increased vegetable consumption and decreased cancer risk in 1993, there has been a growing literature reporting human health benefits of cruciferous vegetables, more specifically, indoles (73, 74). In poultry research, however, there is a lack of information of the effects of any forms of indole supplementation. In addition, the production of indoles, especially food-grade indole naturally derived is very highly priced, due to an expensive chemical conversion process which makes commercial chicken supplementation unfeasible at this phase. Nonetheless, the present study could support the scientific evidences for beneficial effects of indole supplementation in human as well as animal. From the present study, DIM treatment exhibited significantly upregulated Treg cells and IL-10 expression in *Eimeria*-infected chicken while DIM-treated chickens with no *Eimeria* infection did not increase as much as the group infected indicating that DIM likely displays of better effectiveness in *Eimeira*-infected gut rather than those of healthy ones. In the approach to such a drug to prevent/treat chicken gut inflamed by coccidiosis, indoles might have potentials since coccidiosis is considered one of most problematic disease in the poultry industry.

In summary, this is the first evidence to show the effect of dietary indole in reducing intestinal damage induced by coccidiosis in chickens occurs through the regulation of Treg and Th17 cells in the intestine. Because of the lack of immune reagents to detect chicken cytokines related to the T cell response, the study of T cell immunology in chickens has been lagging far behind that in mammals. In this study, we validated that monoclonal antibodies which we previously developed (6, 26) for flow cytometry application could be easily applied to study T cell immune response by determining specific cytokine-expressing T cell phenotypes and in our knowledge, this is the first report to stain chicken lymphocytes with four different fluorescent dyes.

## Author Contributions

WK designed the project, performed the experiments, analyzed the data, and wrote the manuscript. HL supervised the research. WM and HL revised the manuscript.

### Conflict of Interest Statement

The authors declare that the research was conducted in the absence of any commercial or financial relationships that could be construed as a potential conflict of interest.
