# Supplementary figures and images for: Indole Treatment Alleviates Intestinal Tissue Damage Induced by Chicken Coccidiosis Through Activation of the Aryl Hydrocarbon Receptor
Source: Front Immunol. 2019 Mar 26;10:560. doi: 10.3389/fimmu.2019.00560 (PMC6443889; doi:10.3389/fimmu.2019.00560)

## Slide 1
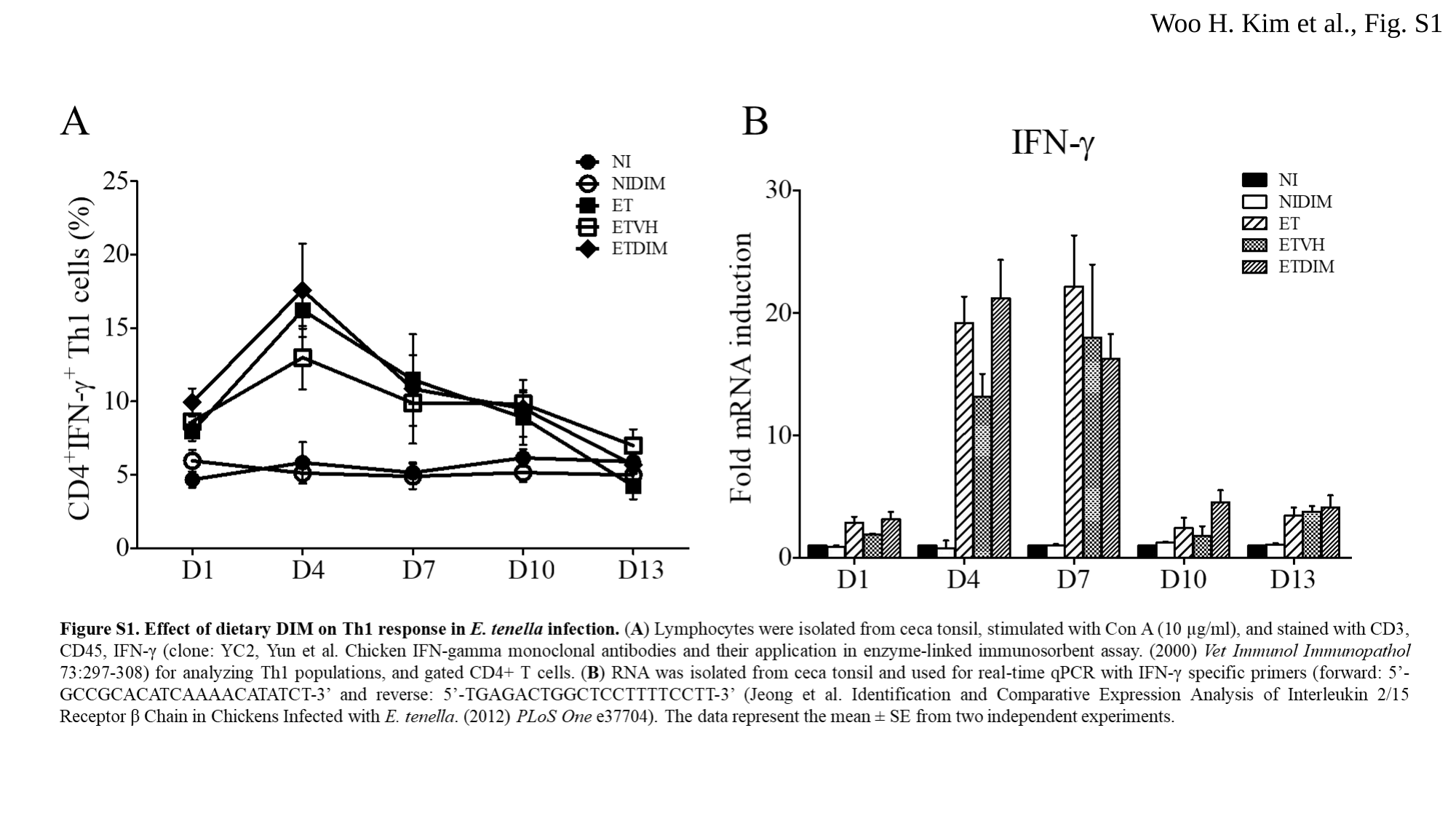

Woo H. Kim et al., Fig. S1

Supplement: Supplementary file 1 [file Presentation_1.PPTX]
